# Should Underweight Donors Be Routinely Procured for Heart Transplantation: A Propensity-Matched Cohort Study

**DOI:** 10.3390/jcm15020799

**Published:** 2026-01-19

**Authors:** Matiullah Masroor, Jing Wang, Yuqi Chen, Yixuan Wang, Cheng Deng, Nianguo Dong

**Affiliations:** 1 Department of Cardiovascular Surgery, Wuhan Union Hospital, Tongji Medical College, Huazhong University of Science and Technology, Wuhan 430022, China; drmasroor@hust.edu.cn (M.M.); 2007xh0994@hust.edu.cn (J.W.); 2024xh8008@hust.edu.cn (Y.C.); wyx135791ab@hust.edu.cn (Y.W.); 2 Department of Cardiothoracic and Vascular Surgery, Amiri Medical Complex, Kabul 1010, Afghanistan

**Keywords:** underweight donors, donor BMI, heart transplantation, survival outcomes, postoperative complications

## Abstract

**Objective**: The impact of underweight or low-BMI donors on heart transplantation (HTx) outcomes remains poorly understood. This study aims to investigate the effect of underweight donors on post-transplant outcomes. **Methods**: We retrospectively analyzed 574 patients divided into 2 groups based on donor BMI: underweight donors (BMI < 20 kg/m^2^, *n* = 101, 17.6%) and normal-weight donors (BMI 20–25 kg/m^2^, *n* = 473, 82.4%). Baseline variables and postoperative outcomes were compared using the Student’s t-test for continuous variables and the chi-squared test for categorical variables. Propensity score matching (PSM) was performed to balance baseline differences and control for confounders. Survival analysis was performed using the Kaplan–Meier method. **Results**: The matched cohort included 71 patients per group, with balanced baseline characteristics. Compared to the normal-weight group, recipients of underweight donors had significantly higher rates of respiratory complications (64.8% vs. 47.9%, *p* = 0.042), neurological complications (15.9% vs. 4.2%, *p* = 0.021), renal complications (17.4% vs. 5.6%, *p* = 0.029), and longer postoperative hospital stay (37.2 vs. 28.4 days, *p* < 0.001). No significant difference was observed in hospital mortality (2.8 vs. 4.2%, *p* = 0.649). The overall follow-up time was 72.2 ± 1.9 months (range 68.5 to 75.8). The 1-, 3-, and 5-year survival rates for the underweight and normal-weight donor BMI groups were 83.1% vs. 85.9% (*p* = 0.624), 75.6% vs. 80.2% (*p* = 0.527), and 72.0% vs. 77.3% (*p* = 0.468), respectively. **Conclusions**: Patients receiving hearts from underweight donors demonstrate comparable long-term survival to those from normal-weight donors but have a higher risk of postoperative complications. These findings suggest that underweight donors could be cautiously utilized to expand the donor pool, offering lifesaving opportunities to recipients who might otherwise experience adverse outcomes due to donor scarcity, albeit with an increased risk of postoperative complications.

## 1. Introduction

Heart transplantation (HTx) remains the gold-standard treatment for end-stage heart failure [1,2]. However, a persistent scarcity of donor organs poses a significant challenge. The shortage has necessitated the use of marginal donors, including those with circulatory death, advanced age, viral infections (e.g, CMV, HBV, HCV, HIV, and COVID-19), cardiac abnormalities (e.g., hypertrophic cardiomyopathy, left ventricular dysfunction), metabolic comorbidities (e.g., hypertension, diabetes mellitus), prolonged ischemic time, a history of drug overdose, and surgically repaired hearts [3,4].

While extreme-BMI donors might be considered suboptimal, research has predominantly focused on the risks associated with donor obesity, leaving the impact of underweight donors poorly characterised. Notably, no study has exclusively examined the impact of underweight donors on heart transplantation. Clear guidelines regarding the indication or contraindication of underweight donor BMI in HTx are lacking, while the current focus is on avoiding donors undersized by weight, BMI, or predicted heart mass (PHM) [5]. This research gap may be due to the relatively small number of underweight populations globally. The International Thoracic Organ Transplant Registry of the International Society of Heart and Lung Transplantation (ISHLT) 2020 annual report on adult HTx, which focused on donor characteristics, also did not discuss underweight donors and their impact on HTx outcomes [6]. While some studies have investigated underweight or low-BMI donors in other transplant contexts, they found that lower donor BMI had no impact on post-transplantation outcomes in lung transplantation [7] or intestinal transplantation [8].

The underweight donor category can be partially represented by lower donor-recipient weight, BMI, or PHM ratios. However, these ratios imperfectly represent this relationship, as obese donors and recipients can still yield ratios < 0.8, and vice versa. Clarifying the relationship of underweight donors is critical, as underweight donors—often younger and with fewer comorbidities—could expand the donor pool without compromising outcomes. Despite growing interest in donor selection criteria, no study has specifically investigated the effect of underweight donor BMI on heart transplant recipients. Our study addresses this critical gap by comparing matched cohorts of heart transplant recipients from underweight versus normal-weight donors, providing the first dedicated analysis of how donor underweight status impacts post-transplant outcomes.

## 2. Materials and Methods

### 2.1. Study Population

Between January 2012 and December 2021, 821 patients underwent HTx at Wuhan Union Hospital. After applying exclusion criteria (patients aged <18 years, re-transplantation, multiorgan transplants, and missing donor BMI data), 653 patients remained eligible. Among these, 79 recipients of overweight or obese donors (BMI ≥ 25 kg/m^2^) were excluded due to limited sample size. The final cohort comprised 574 patients who received grafts from donors with a BMI < 25 kg/m^2^, stratified into 2 groups: those who received grafts from underweight donors (BMI < 20 kg/m^2^, *n* = 101) and those who received grafts from normal-weight donors (BMI 20–25 kg/m^2^, *n* = 473). Due to the small sample size of donors with a BMI < 18.5 kg/m^2^ (*n* = 43), the underweight group was defined as donor BMI < 20 kg/m^2^ to provide a statistically analyzable cohort. To address baseline imbalances, propensity score matching (PSM) was performed, yielding 71 matched pairs (underweight vs. normal-weight) for comparative analysis. The primary outcome was long-term mortality.

### 2.2. Data Collection and Follow-Up

The data were collected from the patient database, which was compiled from the patients’ charts and updated annually based on patient follow-up visits or telephone contact with the patients or their relatives. Patients were followed up at 2 weeks, monthly for the first two months, every two months for the next six months, and annually thereafter.

### 2.3. Donor Heart Procurement and Preservation

After application of the aortic cross clamp, cardioplegic arrest was achieved with 1000 mL of histidine-tryptophan-ketoglutarate (HTK) solution. The donor heart was explanted by resecting the pulmonary veins, superior and inferior vena cava, pulmonary artery, and aorta. An additional 1000–2000 mL of cold (8 °C) HTK solution was infused through the aortic cannula, and the heart was placed in a three-layer sterile plastic bag. The heart was transported submerged in HTK solution, surrounded by ice in a specialized box. Before implantation, another 500 mL of HTK solution was transfused. The left upper and right lower pulmonary veins were marked, and the left atrium was trimmed.

### 2.4. Definition of Terms

Ischemic time: The total cold and warm ischemic time of the donor heart, from the application of the aortic cross-clamp in the donor to its removal in the recipient after aortic anastomosis.

Respiratory complications: Defined as pulmonary infection (e.g., pneumonia), pleural effusion, or the requirement for tracheostomy or prolonged endotracheal intubation due to respiratory failure.

Neurological complications: Defined as a transient ischemic attack or ischemic/hemorrhagic stroke diagnosed by CT or clinical presentation.

Renal complications: Defined as new-onset acute kidney injury diagnosed by laboratory and clinical criteria, or the requirement for continuous renal replacement therapy.

The diagnoses of these complications were made by the ICU physicians, and the data for the analysis were collected retrospectively from the patient dataset.

### 2.5. Statistical Analysis

Continuous variables are presented as mean ± standard deviation and were compared using the independent Student’s *t*-test. Categorical variables are expressed as numbers (percentages) and were analyzed using the chi-square test. PSM was performed to balance baseline characteristics between groups, incorporating variables that showed significant differences in the unmatched cohort. A 1:1 nearest-neighbour matching was applied with a caliper width of 0.1. Survival analysis was conducted using the Kaplan–Meier method, with between-group comparisons assessed via the log-rank test. A *p*-value < 0.05 was considered statistically significant for all analyses.

## 3. Results

### 3.1. Baseline Patient Characteristics

Following propensity score matching, the mean recipient age was comparable between groups (47.1 years vs. 47.9 years, *p* = 0.753). Underweight donor recipients (BMI < 20 kg/m^2^) constituted 17.6% (101/574) of the cohort, while the normal-weight donor recipients constituted 82.4% (473/574). As expected, donor BMI differed significantly between groups (18.5 ± 1.4 kg/m^2^ vs. 22.2 ± 1.4 kg/m^2^, *p* < 0.001), validating the group stratification. Prior to matching, several recipient characteristics differed significantly, including sex, weight, height, BMI, PHM, heart failure etiology, and smoking history. Significant differences were also observed in donor variables, including age, sex, and cause of death. All variables were balanced after matching, except for donor weight, height, BMI, PHM, and donor-recipient weight, BMI, and PHM ratio, which were inherently different due to the group definitions. To minimize the influence of sex on outcomes, a combined donor-recipient sex variable was created. Sex matching was significantly different between groups before matching, but was balanced after PSM. Demographic and preoperative variables are shown in Table 1.

### 3.2. Postoperative Outcomes

Before matching, the two donor BMI groups showed no significant differences in most postoperative outcomes, except for ICU stay and neurological complications. Neurological complications were more frequent in the underweight donor group (14.3% vs. 6.4%, *p* = 0.019), while ICU stay was slightly longer in the normal-weight donor group (10.4 days vs. 9.8 days, *p* = 0.024). After matching, the underweight donor cohort demonstrated significantly higher rates of postoperative complications: respiratory complications (64.8% vs. 47.9%, *p* = 0.042), also reflected by positive sputum culture (55.1% vs. 35.2%, *p* = 0.018); neurological complications (15.9% vs. 4.2%, *p* = 0.021); and renal complications (17.4% vs. 5.6%, *p* = 0.029). The difference in ICU stay became nonsignificant after matching, but the total hospital stay remained significantly longer for recipients of underweight donors (37.2 days vs. 28.4 days, *p* < 0.001). The prolonged hospital stay was likely a consequence of the higher complication rate. Linear logistic regression analysis showed that respiratory complications prolonged hospital stay by 9 days (standardized coefficient, *B* = 9.5, *p* < 0.001), renal complications by 11 days (*B* = 10.9, *p* = 0.001), and neurological complications by 12 days (*B* = 12.7, *p* = 0.005). Hospital mortality rates were comparable between groups (2.8% vs. 4.2%, *p* = 0.649), as was the incidence of acute graft rejection (1.4% in both groups). All other postoperative outcomes, including cardiopulmonary bypass time, total surgery duration, and mechanical circulatory support requirements, showed no significant differences (Table 2).

### 3.3. Survival Outcomes

**Figure 1:**
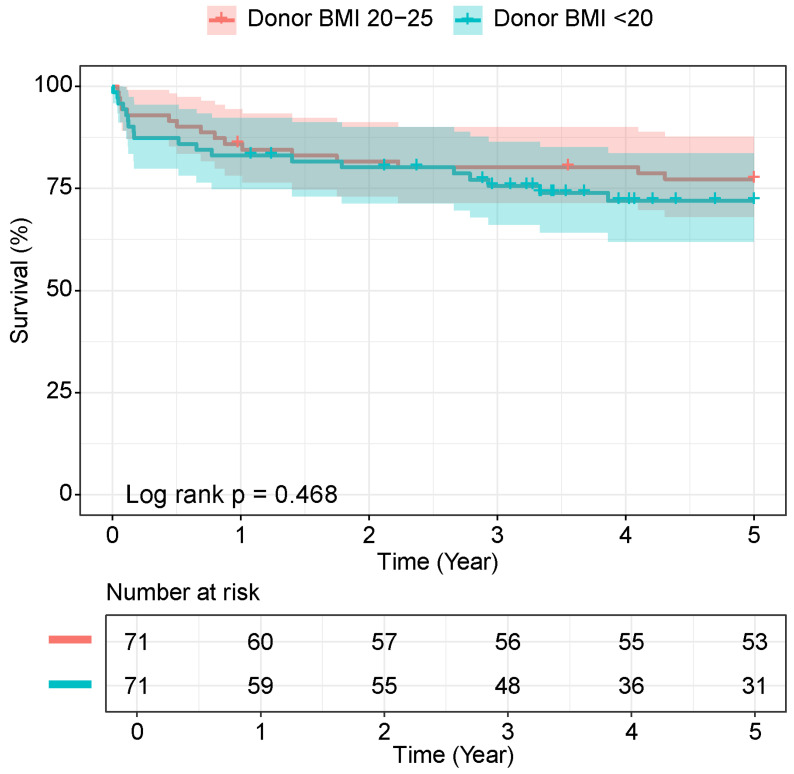
The Kaplan–Meier survival curve of the two donor BMI-based groups.

The overall mean follow-up time was 72.2 ± 1.9 months (range 68.5 to 75.8). The mean follow-up time was 62.4 ± 2.9 months for the underweight donor group and 81.4 ± 1.7 months for the normal-weight group. The mean survival time was 70.8 months in the donor BMI < 20 kg/m^2^ group compared to 74.6 months in the BMI 20–25 kg/m^2^ group for the entire cohort. When truncated at 5 years, the mean survival time was 47.3 months and 49.5 months, respectively. Kaplan–Meier analysis demonstrated no statistically significant difference in mortality between groups (log-rank *p* = 0.468), as shown in Figure 1.

Due to substantial censoring beyond 5 years, the survival analysis focused on this timeframe. The 1-, 3-, and 5-year survival rates for the underweight versus normal-weight groups were 83.1% vs. 85.9% (*p* = 0.624), 75.6% vs. 80.2% (*p* = 0.527), and 72.0% vs. 77.3% (*p* = 0.468), respectively. We also compared the two groups using the BMI cutoff of <18.5 kg/m^2^ (*n* = 43 vs. 531). The results showed no significant difference in survival (log-rank *p* = 0.300) or postoperative outcomes, as shown in the Supplementary Figure S1 and Supplementary Table S1. The underweight group using the <18.5 cutoff should have shown even worse outcomes compared to the previously used cutoff. We attribute this lack of significance primarily to the loss of statistical power from the small sample size (*n* = 43) and, most importantly, baseline imbalances that could not be corrected with PSM due to the limited number of patients.

## 4. Discussion

This study provides the first dedicated analysis of heart transplantation outcomes using underweight donors (BMI < 20 kg/m^2^), a population largely overlooked in the existing literature. Our findings demonstrate that while underweight donors yield comparable long-term survival, they are associated with increased postoperative complications, highlighting both the potential to expand the donor pool and the need for cautious postoperative management.

While prior research has examined the risks obese donors pose to patient and graft survival, the impact of low donor BMI has remained unexplored. Alyadin et al. [9] studied the relationship between donor BMI and post-transplantation survival by dividing patients into survivor and non-survivor groups. They discovered that recipients in the non-survivor group were more likely to have received hearts from obese donors. Conversely, Shudo et al. [10] reported no significant differences in short-term outcomes between recipients of hearts from donors with higher BMI and those with normal BMI. This indicates that donor obesity may not universally predict adverse outcomes. Krebs et al. [11] expanded on this by analyzing data from the UNOS database between 2003 and 2017. They categorized donors based on BMI thresholds of ≥40 kg/m^2^ and <40 kg/m^2^ and found that donor obesity has no negative impact on post-transplantation outcomes. Similarly, Kim et al. [12] analyzed UNOS data from 2000 to 2020, using propensity score matching to compare outcomes based on donor obesity. They also evaluated the effect of donor ischemic time, categorizing cases into those with ischemic time <4 h and ≥4 h. They observed no difference in 30-day mortality or 5-year survival when ischemic time was <4 h, regardless of donor BMI. Interestingly, in cases with ischemic times exceeding 4 h, recipients of hearts from obese donors showed better outcomes than those who received hearts from normal-weight donors [12]. What is notably absent from these investigations is any focused examination of underweight donors.

We could not retrieve any prior research specifically on the effect of underweight donors in HTx. This research gap likely reflects the obesity pandemic and its association with cardiovascular diseases [13], with more than 71% of the US population being overweight or obese recently, and more than 50% of the world population will be overweight or obese by 2035 [14]. We believe the obesity pandemic has directed scientific attention toward studying obese donors rather than underweight ones. According to the 2022 WHO data, the prevalence of the underweight population (BMI < 18.5) is 1.7% in the USA, while 4.1% in China, explaining the limited underweight donor pool. In our cohort, the underweight donors (BMI < 20 kg/m^2^) constituted 17.6%, while only 7.5% had a BMI < 18.5 kg/m^2^. The only remotely relevant study on underweight donors used the UNOS registry data from 2019 to 2024 and combined underweight and normal-weight donors (BMI < 25 kg/m^2^), preventing a specific assessment of the underweight group. Their study showed no survival difference at 1 month, 1 year, and 5 years, *p* = 0.825, compared to the other groups. The rate of postoperative complications such as stroke, retransplantation, and graft survival at 1 and 5 years was also not significantly different [15]. The mean donor BMI of the combined normal/underweight group was 22.58, which is similar to our normal-weight group. Our study also found comparable survival outcomes at 1-, 3-, and 5-year in both the unmatched and matched cohorts. But some postoperative complications, such as respiratory, renal, and neurological, were higher in the underweight donor group after matching. The postoperative hospital stay in the underweight group was longer than the normal-weight group (37.2 ± 17.6 vs. 28.4 ± 13.3, *p* < 0.001). We believe the longer hospital stay was caused by the higher complication rate in this group, which needed extra care. Respiratory complications prolonged the hospital stay by 9 days, renal complications by 11 days, and neurological complications by 12 days. Acute rejection and hospital mortality were not statistically different between the groups. Studies of the underweight donors in liver transplantation also found no differences in outcomes [7]. A UNOS database analysis for intestinal transplantation also did not find any significant difference in survival among different donor BMI groups [8].

The underweight donor category can be partially represented by lower donor-recipient weight, height, BMI, BSA, and PHM ratios. PHM is considered the most promising metric for donor-recipient size matching, with a ratio < 0.86 linked to unfavorable outcomes [5]. However, an underweight donor with a BMI < 18.5 kg/m^2^ can have a normal donor-recipient PHM ratio for a given recipient and vice versa, meaning these ratios do not fully represent the underweight donor category. The donor PHM and the donor-recipient PHM ratio in our study can help us understand whether the higher complications in the underweight group were caused by donor undersizing. Although the donor PHM and PHM ratio in the underweight group was significantly smaller than the normal-weight group (137 ± 18 vs. 157 ± 20, *p* < 0.001) and (0.96 ± 0.2 vs. 1.07 ± 0.2, *p* = 0.002), the PHM ratio was very close to 1 and above the unfavorable threshold. This suggests that the higher complications in the underweight group of this study were not primarily driven by significant undersizing as defined by PHM, although undersizing by PHM can indeed have worse outcomes. To explore the effect of PHM in the underweight donor group, a large registry dataset is required with enough sample size, which allows for stratification of this group based on PHM. Additionally, the D-R weight and BMI ratios, though statistically significantly smaller in the underweight group, were still above the cutoff value of 0.8.

Because of the very low potential underweight donor pool, we do not have data regarding their outcomes in HTx. But we still have underweight heart donors, as represented by our study, and the better utilization of these donors can increase the donor pool. Therefore, it is important to study the outcomes of underweight donors after HTx. Given the scarcity of data, this study can be a milestone for outcomes as well as a guide for future studies. Our results demonstrate that underweight donor status should not be a contraindication for heart transplantation, as evidenced by comparable 5-year survival (72.0% vs. 77.3%, *p* = 0.468). However, the significantly higher rates of respiratory (64.8% vs. 47.9%, *p* = 0.042), neurological (15.9% vs. 4.2%, *p* = 0.021), and renal (17.4% vs. 5.6%, *p* = 0.029) complications suggest these patients require special consideration. These findings may reflect physiological stress from smaller allografts, potentially causing hemodynamic instability, or metabolic reserve limitations from possible malnutrition-associated issues.

The absence of prior studies and clinical guidelines on underweight donors represents a critical gap in donor selection guidelines. Heart transplantation centers may be hesitant to use underweight donors due to clinical uncertainty in the absence of evidence, potentially discarding viable organs, or including them without knowing the survival outcomes. Our findings echo the limited data on liver and intestinal transplantation, where underweight donors have been successfully utilized. Notably, the comparable hospital mortality (2.8% vs. 4.2%, *p* = 0.649) suggests that complications, while more frequent in the underweight group, are manageable in experienced centers. Our study provides evidence that hearts from underweight donors yield excellent long-term survival. By quantifying the risk (higher manageable complications) against the benefit (comparable survival), we provide a rationale for the confident and selective use of these donors. This evidence-based approach can thus help expand the potential donor pool by reducing ambiguity and reassuring clinicians.

## 5. Limitations

Several limitations of this study warrant consideration. First, as a single-center investigation, our findings may have limited generalisability due to potential ethnic variations and institution-specific clinical practices. Second, while PSM balances measurable confounders, the relatively small matched cohort may be underpowered to detect differences in rare outcomes. Third, our analysis could not account for all potential confounding variables, and some unmeasured variables, such as primary graft dysfunction, may affect the outcomes. Fourth, the BMI threshold of <20 kg/m^2^, while clinically pragmatic, represents an arbitrary cutoff, and future studies should explore narrower BMI stratification (e.g., <18.5 kg/m^2^ vs. 18.5–20 kg/m^2^).

Validation of these findings through multicenter prospective studies and large sample analyses of international registries (e.g., UNOS/ISHLT) is essential. Furthermore, mechanistic studies exploring the pathophysiology behind the increased complication risks, including analyses of inflammatory cytokine profiles, cardiac biomechanical properties, and metabolic reserve in underweight donors, could provide valuable biological insights to complement these clinical observations.

## 6. Conclusions

This study demonstrates that hearts from underweight donors (BMI < 20 kg/m^2^) represent a clinically viable option for transplantation, achieving comparable long-term survival to normal-weight donors. However, the significantly higher incidence of respiratory, neurological, and renal complications necessitates judicious donor selection and vigilant postoperative care.

These findings support the selective use of underweight donors, particularly for high-risk recipients who may derive greater benefit from earlier transplantation despite increased perioperative risks. As organ demand escalates, clarifying and expanding donor selection criteria to include underweight individuals could save lives without compromising long-term outcomes.

## Figures and Tables

**Table 1:** Baseline demographic and preoperative variables of the two donor BMI-based cohorts before and after matching.

	Unmatched			Matched		
Variables	Donor BMI <20 kg/m^2^ (*n* = 101)	Donor BMI 20–25 kg/m^2^ (*n* = 473)	*p*-Value	Donor BMI <20 kg/m^2^ (*n* = 71)	Donor BMI 20–25 kg/m^2^ (*n* = 71)	*p*-Value
Recipient age (years)	46.0 ± 14.4	48.2 ± 11.8	0.103	47.1 ± 14.9	47.9 ± 12.7	0.753
Recipient sex (male) (%)	67 (66.3)	386 (81.6)	**<0.001**	47 (66.2)	50 (70.4)	0.588
Recipient weight (kg)	60.6 ± 12.2	65.0 ± 12.7	**0.002**	61.1 ± 11.2	63.0 ± 11.5	0.316
Recipient height (cm)	165.0 ± 8.0	167.9 ± 7.2	**<0.001**	165.4 ± 6.9	167.2 ± 6.9	0.133
Recipient BMI (kg/m^2^)	22.1 ± 3.6	22.9 ± 3.7	**0.047**	22.3 ± 3.4	22.5 ± 3.3	0.729
Recipient PHM	146 ± 28	157 ± 26	**<0.001**	147 ± 26	151 ± 26	0.322
Diagnosis (%)			**0.003**			0.919
NICM	68 (67.3)	295 (62.4)		47 (66.2)	48 (67.6)	
ICM	11 (10.9)	109 (23.0)		10 (14.1)	12 (16.9)	
VHD	10 (9.9)	48 (10.1)		7 (9.9)	6 (8.5)	
CHD	7 (6.9)	16 (3.4)		4 (5.6)	2 (2.8)	
Others	5 (5.0)	5 (1.1)		3 (4.2)	3 (4.2)	
Ischemic time (min)	317 ± 129	321 ± 113	0.780	336 ± 114	346 ± 134	0.644
Donor age (years)	30.6 ± 12.6	36.6 ± 11.0	**<0.001**	31.1 ± 13.1	33.7 ± 12.2	0.218
Donor sex (male) (%)	75 (76.5)	415 (90.8)	**<0.001**	52 (73.2)	57 (80.3)	0.320
Donor-recipient sex (%)			**<0.001**			0.768
Male-Male	52 (52.0)	347 (75.9)		37 (52.1)	42 (59.2)	
Male-Female	24 (24.5)	68 (14.9)		15 (21.1)	15 (21.1)	
Female-Male	13 (13.3)	24 (5.3)		10 (14.1)	8 (11.3)	
Female-Female	10 (10.2)	18 (3.9)		9 (12.7)	6 (8.5)	
Donor weight (kg)	50.9 ± 5.7	64.1 ± 6.0	<0.001	50.8 ± 5.7	63.1 ± 6.7	<0.001
D-R weight ratio	0.87 ± 0.20	1.02 ± 0.21	<0.001	0.85 ± 0.17	1.03 ± 0.21	<0.001
Donor height (cm)	165.7 ± 6.3	169.0 ± 5.2	<0.001	165.4 ± 6.4	168.5 ± 5.9	0.003
Donor BMI (kg/m^2^)	18.5 ± 1.4	22.4 ± 1.4	<0.001	18.5 ± 1.4	22.2 ± 1.4	<0.001
D-R BMI ratio	0.86 ± 0.15	1.0 ± 0.17	<0.001	0.85 ± 0.15	1.0 ± 0.16	<0.001
Donor PHM	138 ± 17	161 ± 15	<0.001	137 ± 18	157 ± 20	<0.001
D-R PHM ratio	0.98 ± 0.2	1.05 ± 0.2	<0.001	0.96 ± 0.2	1.07 ± 0.2	0.002
Hx of cardiac surgery (%)	23 (33.8)	93 (30.5)	0.591	21 (33.9)	18 (25.7)	0.305
Hx of hypertension (%)	9 (12.9)	58 (17.4)	0.325	8 (12.7)	11 (15.7)	0.620
Hx of diabetes (%)	9 (15.0)	61 (21.7)	0.243	8 (14.5)	9 (13.0)	0.809
Hx of hyperlipidemia (%)	3 (5.2)	13 (4.9)	0.923	2 (3.8)	4 (5.8)	0.608
Hx of stroke	2 (3.4)	10 (4.1)	0.816	2 (3.8)	1 (1.5)	0.419
Hx of CKD (%)	4 (5.1)	21 (5.8)	0.824	4 (5.6)	1 (1.4)	0.172
Hx of dialysis (%)	2 (2.5)	8 (2.2)	0.851	2 (2.1)	1 (1.4)	0.560
Hx of CLD (%)	7 (9.0)	24 (6.6)	0.451	6 (8.5)	1 (1.4)	0.053
HX of PVD (%)	3 (3.9)	10 (2.7)	0.588	3 (4.3)	1 (1.4)	0.304
Hx of smoking (%)	20 (25.6)	163 (44.7)	**0.002**	19 (26.8)	20 (28.2)	0.851
Hx of alcoholism (%)	12 (16.7)	92 (27.0)	0.164	11 (16.9)	13 (18.6)	0.802
Creatinine (μmol/L)	97 ± 57	100 ± 49	0.613	98 ± 59	89 ± 29	0.214
Total bilirubin (μmol/L)	24.9 ± 19.7	28.3 ± 23.4	0.230	24.8 ± 20.1	26.0 ± 21.2	0.712
Albumin (g/L)	39.8 ± 5.6	39.1 ± 4.5	0.237	39.8 ± 5.5	40.0 ± 4.0	0.722
Hemoglobin (g/L)	132 ± 22	133.6 ± 22	0.755	132 ± 22	131.6 ± 23	0.955
HX of IABP (%)	1 (1.3)	9 (2.5)	0.516	1 (1.4)	0 (0.0)	0.316
Hx of ECMO (%)	2 (2.5)	6 (1.6)	0.588	2 (2.8)	0 (0.0)	0.154
Preop ventilation (%)	2 (2.5)	13 (3.6)	0.649	2 (2.8)	0 (0.0)	0.154
Ejection fraction (%)	29.9 ± 13.2	27.6 ± 10.9	0.068	29.5 ± 13.5	29.8 ± 12.8	0.889
LV diameter (cm)	6.6 ± 1.9	7.4 ± 4.2	0.085	6.6 ± 1.8	7.0 ± 1.6	0.187
Cause of donor death (%)			**0.006**			0.782
Traumatic brain injury	64 (66.0)	278 (61.1)		44 (62.0)	44 (62.0)	
Cerebrovascular disease	18 (18.6)	140 (30.8)		15 (21.1)	18 (25.4)	
Brain tumor	3 (3.1)	18 (4.0)		2 (2.8)	2 (2.8)	
Anoxic brain death	9 (9.3)	15 (3.3)		9 (12.7)	5 (7.0)	
Else	3 (3.1)	4 (0.9)		1 (1.4)	2 (2.8)	

PHM: Predicted heart mass, NICM: Non-ischemic cardiomyopathy, ICM: Ischemic cardiomyopathy, VHD: Valvular heart disease, CHD: Congenital heart disease, Hx: History, CKD: Chronic kidney disease, CLD: Chronic liver disease, PVD: Peripheral vascular disease, LV: Left ventricle.

**Table 2:** Postoperative outcomes of the two donor BMI-based cohorts before and after matching.

	Unmatched			Matched		
Variables	Donor BMI <20 kg/m^2^ (*n*= 101)	Donor BMI 20–25 kg/m^2^ (*n* = 473)	*p*-Value	Donor BMI <20 kg/m^2^ (*n* = 71)	Donor BMI 20–25 kg/m^2^ (*n* = 71)	*p*-Value
CPB time (min)	113 ± 33	117 ± 57	0.263	112 ± 33	115 ± 49	0.585
Cross clamp time (min)	32 ± 9	32 ± 9	0.459	32 ± 10	30 ± 9	0.149
Surgery time (min)	257 ± 84	269 ± 92	0.145	255 ± 82	249 ± 73	0.620
Postop ventilation (hours)	58.8 ± 77	72.7 ± 171	0.149	69 ± 89	67 ± 131	0.898
Hospital mortality (%)	2 (2.5)	22 (6.0)	0.214	2 (2.8)	3 (4.2)	0.649
ICU stay (days)	9.8 ± 5.5	10.4 ± 9.6	**0.024**	10.7 ± 5.9	11.7 ± 7.7	0.390
Acute rejection (%)	1 (1.3)	7 (1.9)	0.695	1 (1.4)	1 (1.4)	>0.999
Respiratory complication (%)	51 (64.6)	223 (61.1)	0.566	46 (64.8)	34 (47.9)	**0.042**
Neurological complication (%)	11 (14.3)	23 (6.4)	**0.019**	11 (15.9)	3 (4.2)	**0.021**
Renal complication (%)	13 (16.9)	61 (17.0)	0.982	12 (17.4)	4 (5.6)	**0.029**
Septic shock (%)	0 (0.0)	13 (4.6)	0.074	0 (0.0)	2 (2.9)	0.187
Positive blood culture (%)	8 (11.1)	49 (14.5)	0.456	8 (12.1)	5 (7.1)	0.324
Positive sputum culture (%)	40 (53.3)	185 (53.0)	0.959	38 (55.1)	25 (35.2)	**0.018**
Postop EF (%)	65.7 ± 5.3	64.8 ± 6.9	0.283	65.4 ± 5.3	65.3 ± 7.6	0.915
Postop IABP (%)	30 (29.7)	167 (35.3)	0.282	26 (36.6)	27 (38.0)	0.862
Postop ECMO (%)	5 (5.0)	30 (6.3)	0.596	4 (5.6)	5 (7.0)	0.731
Postop CRRT (%)	9 (9.1)	66 (14.2)	0.175	8 (11.4)	6 (8.5)	0.554
Postop hospital stay (days)	35.5 ± 16.5	37.7 ± 20.0	0.067	37.2 ± 17.6	28.4 ± 13.3	**<** **0** **.001**

CPB: Cardiopulmonary bypass, EF: Ejection fraction, IABP: Intra-aortic balloon pump, ECMO: Extra corporeal membrane oxygenation, CRRT: Continuous renal replacement therapy.

## Data Availability

The original contributions presented in this study are included in the article. Further inquiries can be directed to the corresponding authors.

## Supplementary Materials

The following supporting information can be downloaded at: https://www.mdpi.com/article/10.3390/jcm15020799/s1, Figure S1: KM survival curve of the donor BMI-based cohort; Table S1: Postoperative outcomes of the two donor BMI-based cohorts.
