# Supplementary material for: Should Underweight Donors Be Routinely Procured for Heart Transplantation: A Propensity-Matched Cohort Study
Source: J Clin Med. 2026 Jan 19;15(2):799. doi: 10.3390/jcm15020799 (PMC12841971; doi:10.3390/jcm15020799)
Supplement: Supplementary file 1 [file jcm-15-00799-s001.zip › jcm-4062315-supplementary.pdf]

Supplementary data

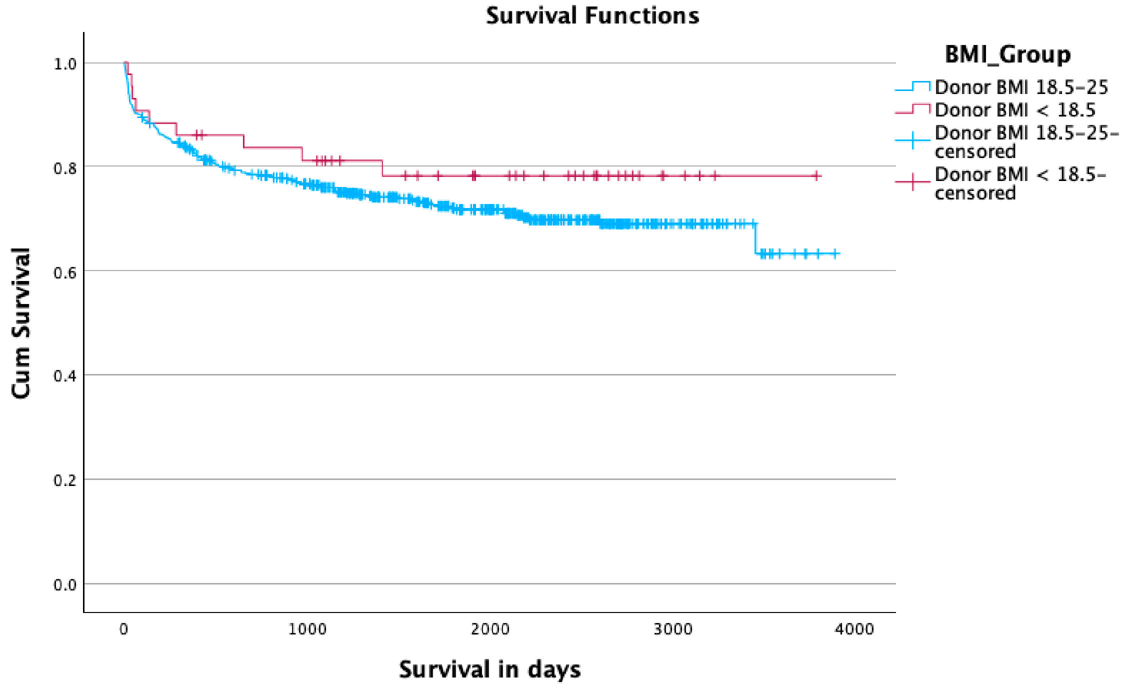


Supp Figure S1. KM survival curve of the donor BMI-based cohort (BMI <18.5 vs. 18.5-25). (Log-rank *p = 0.3000*

Supp Table S1. Postoperative outcomes of the two donor BMI-based cohorts

| Variables | Donor BMI <18.5 kg/ m$^2$ (*n*= 43) | Donor BMI 18.5-25 kg/ m$^2$ (*n*= 531) | *p*-value |
|---|---|---|---|
| CPB time (min) | 115±33 | 116±55 | 0.902 |
| Cross clamp time (min) | 34±12 | 32±9 | 0.240 |
| Surgery time (min) | 271±87 | 266±91 | 0.755 |
| Postop ventilation (hours) | 57.6±70 | 71.2±164 | 0.589 |
| Hospital mortality (%) | 0(0.0) | 24(5.8) | 0.147 |
| ICU stay (days) | 9.8±5.4 | 10.3±9.3 | 0.762 |
| Acute rejection (%) | 0(0.0) | 8(1.9) | 0.412 |
| Respiratory complication (%) | 22(64.7) | 252(61.5) | 0.709 |
| Neurological complication (%) | 5(15.2) | 29(7.2) | 0.100 |
| Renal complication (%) | 6(18.2) | 68(16.9) | 0.847 |
| Septic shock (%) | 0(0.0) | 13(4.0) | 0.279 |
| Positive blood culture (%) | 4(13.3) | 53(13.9) | 0.930 |

| | | | |
|---|---|---|---|
| Positive sputum culture (%) | 12(38.7) | 213(54.2) | 0.096 |
| Postop EF (%) | 65.3±5.7 | 65±6.7 | 0.738 |
| Postop IABP (%) | 12(27.9) | 185(34.8) | 0.357 |
| Postop ECMO (%) | 2(4.7) | 33(6.2) | 0.680 |
| Postop CRRT (%) | 4(9.8) | 71(13.6) | 0.488 |
| Postop hospital stay (days) | 35.3±13.6 | 37.4±19.8 | 0.510 |
